# Enabling Transformation of Food Systems: Some Issue to Address

**DOI:** 10.1016/j.cdnut.2024.104463

**Published:** 2024-09-26

**Authors:** Eileen Kennedy, Rosemary Green

**Affiliations:** 1 Friedman School of Nutrition Science and Policy, Tufts University, Boston, MA, United States; 2 London School of Hygiene and Tropical Medicine, London, United Kingdom

There is a clarion call for a dramatic transformation of the world's diets [1]. At present, 1 in 3 people are malnourished [2]. This includes persistent undernutrition in many countries, but also a burgeoning global obesity epidemic that often coincides with micronutrient malnutrition, leading the United Nations to define “malnutrition in all its forms” as a pressing health concern. The composition of diets has changed, in part due to the urbanization of households and changing food environments. Traditional diets based on staple grains with limited variety have been replaced increasingly by much more diverse diets with more sugar, fat, and sodium [3]. Indeed, poor diets are the leading cause of poor health worldwide according to the Global Burden of Disease study [4].

Diets and food systems are inextricably connected with each other, and for diets to improve, there must be transformation across the food system from agriculture, through supply chains to food environments, consumption, and waste (Figure 1). Food systems have developed to focus on quantity rather than quality of food, and in doing so they have enabled huge population expansion, but frequently at the expense of nutritional adequacy and sustainability. Without purposeful action we will fall short of achieving any of the 17 Sustainable Development Goals (SDGs) for food security, nutrition, and sustainable agriculture [5]; the world is calling for dramatic transformation of our food systems to address the challenges embedded in the SDGs. Food systems affect everyone.

**FIGURE 1:**
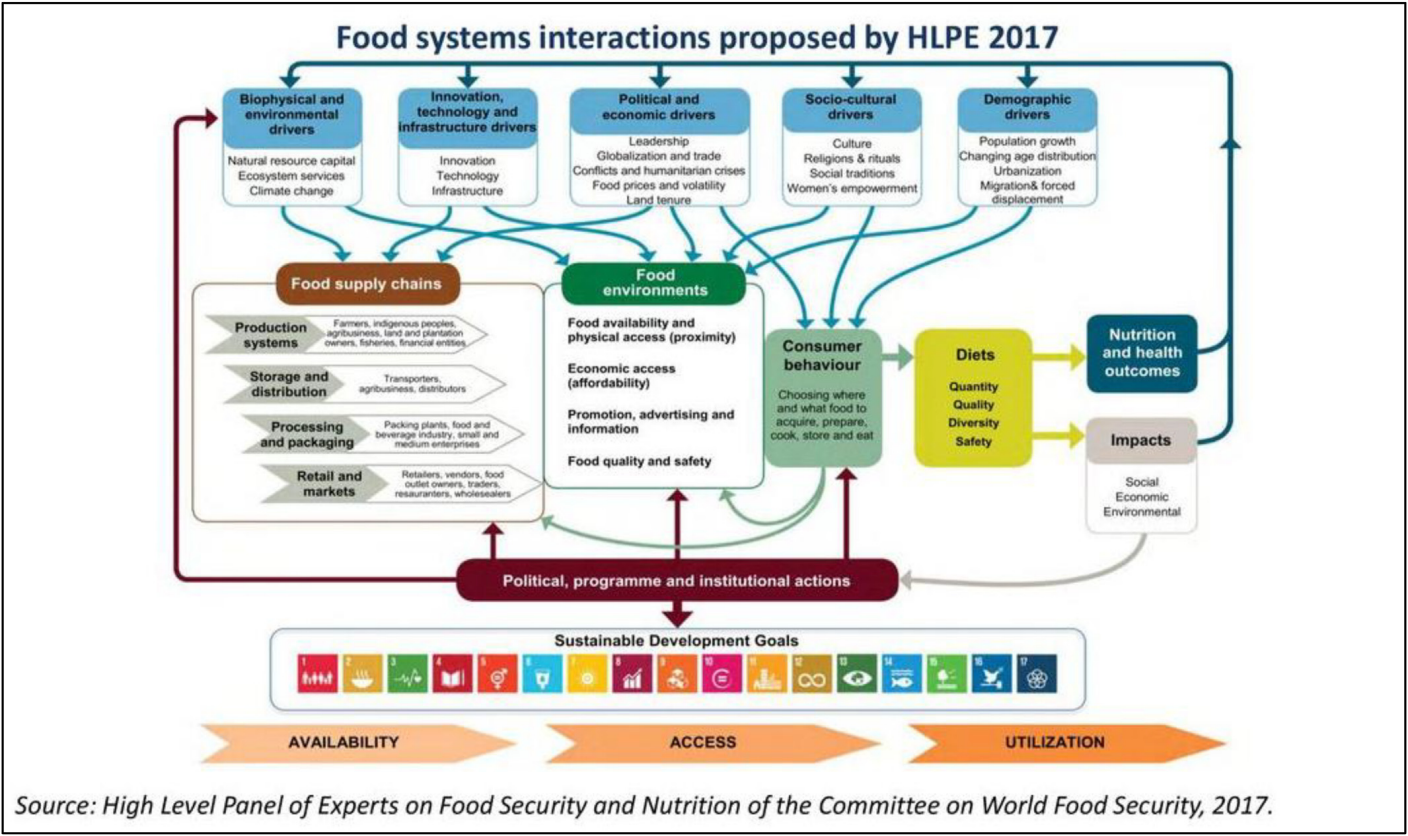
Food systems interactions [1].

In addition to damaging our health, food systems around the world are also damaging our natural environment. Food is responsible for up to a third of global greenhouse gas emissions, 70% of water use, and the vast majority of biodiversity loss [1]. In fact, recent analyses have shown that the world cannot meet the targets of the Paris Agreement without reducing emissions from our food systems [6]. Environmental impacts can be reduced by changing food production methods (for example, by managing use of fertilizer and water carefully), but dietary change can also play a major part because animal-source foods are responsible for the majority of emissions, land and water use.

The United Nations Food Systems Summit in 2021 placed a bright spotlight on the need to transform food systems to better ensure environmental and human health [7]. The global transformation of food systems is expected to increase the sustainability and climate resilience of agricultural production, ensure healthy and safe diets, and contribute to healthy lifestyles. The task may seem onerous.

There are, however, opportunities to make changes in current food systems that improve dietary patterns and nutrition in all countries. Several of these opportunities are discussed below.

## Food Environments

Food environments are defined as the physical, economic, and sociocultural context in which consumers engage with the food system to make decisions about acquiring, preparing, and consuming food [1]. The structure of food environments has a major effect on the availability, physical access, affordability, and desirability of foods. There is wide variability in food environments across countries, and even within a particular nation. Not surprisingly given the panoply of food environments, efforts for improvement have varied. As countries industrialize, the food environment becomes more complex and no longer includes simply farm-level and village-level sources of foods. In a complicated food environment, efforts focused on the availability of foods may be in contrast to efforts to increase access. For example, strategies to increase the availability of fruits and vegetables by investing in cold chains may not simultaneously increase access to these products if purchase price of these products is increased.

The price of food can be a barrier to the purchase of nutritious foods. On the basis of established criteria, data from a multicountry study show that about 3 billion people cannot afford an adequate diet [8].

The composition of diets has also changed, in part due to urbanization of households and changing food environments. This can be a positive development when more nutrient-dense foods are added to the diet, such as fruits, vegetables, nuts, and seeds. Nutrient rich foods, on average, are more expensive than nutrient sparse foods [9].

The need for true cost accounting (TCA) in food systems was highlighted in the concluding Statement of Action from the United Nations Food Systems Summit [7], which noted that “the value of food must be realized, and the economic, social, and environmental impact and externalities must be better assessed and mitigated or leveraged as required.” This statement was reinforced by a series of articles and events preceding the Summit including the Scientific Group’s June 2021 report on True Cost and True Price of Food [10] and the Rockefeller Foundation’s assessment of the true cost of the United States food system [11]. Building on these significant reports, the United Nations Food Systems Summit has provided the momentum to advance knowledge that translates theory into practice related to TCA, a method that accounts for all costs and benefits [7].Although TCA is in its infant stages, its use has potential in facilitating a transition to sustainable, healthy food systems.

## Consumer Behavior

Consumer behavior is difficult to change. A number of factors influence consumer food choice including income, price of food, availability of food, taste, and culture to name a few. As noted by Tewodros et al. [12], there is limited research on specific approaches and behavior change techniques that have been used. Traditionally nutrition education and/or behavior change campaigns targeted to households and/or individuals have been employed to improve dietary behaviors. There are, however, strategies for improving diets and nutrition that have been used that link agriculture to nutritional improvements.

Since the release of the SDGs, there has been renewed interest in agriculture-nutrition linkages. This approach emphasizes changes in agriculture that can promote improved nutrition. Meta-analyses of a range of agriculture-nutrition linkages strategies that have improved diets and nutrition are disappointingly scarce [13]. There are some exceptions.

A multipronged strategy to increase production and consumption of orange-fleshed sweet potato showed that the project increased household production and consumption of sweet potatoes in preschool-aged children [14].

Results from research in Malawi called the Egg Hub Model worked with smallholder farmers to increase egg production, enhance availability, and improve consumption [15]. Egg production tripled, and consumption of eggs increased from 2 to 9 eggs/mo. Importantly, the egg hub model was able to be replicated in Ethiopia, Peru, and Brazil.

Both the orange sweet potato and egg hub examples are currently relevant because they provide effective techniques for linking the agriculture and consumer parts of the food system continuum.

## Data-Metrics

In the activities leading up to the Food Systems Summit, as well as the Summit itself, there was a consistent call for more appropriate metrics to effectively monitor and evaluate the process and impact of transforming food systems. The complex needs for information including the proper type of data, utility of various indicators, relationships across measures, changes over time, the level of precision and accuracy to ensure reliability, completeness, weighting, interpretation were flagged as critical necessities. The articulated list of needs was seen by many as overwhelming.

A significant body of research will be required to address these complex essentials. The UNFSS expressed a concern that novel metrics may be needed, in part, because some of the current, routine measures may be unsatisfactory. One example can be used to illustrate this point.

Diet diversity, or the number of food groups or the number of individual foods, is often used to monitor and evaluate diet quality [16]. Although this measure has been validated and highly recommended due the relative simplicity of data collection schemes, the diet diversity scores as currently constructed may be insufficient as low- and middle-income countries undergo increased urbanization. Urban households are increasing the number of food groups and/or number of foods eaten, but not the kind intended to generate positive health impacts. Diet diversity can be good or bad depending on the form it takes in household diets. A new way of measuring diet diversity by modifying current methods or alternative indicators may need to be developed. The ease of implementation and analysis for any alternative metrics will be key to evaluating the utility of new indicators. For example, research studies routinely use a 24-h recall methodology to measure household/individual food intake information; these data require greater time to collect and analyze than does diet diversity.

A narrow set of indicators has been used to evaluate food systems. More is now needed. Food systems are expected to do more including guaranteeing sustainably produced, affordable foods that are produced in a way that respects the natural environment, providing a living wage, generating increased employment both in rural areas and the burgeoning urban areas in many countries, and advancing equity and social justice. What are more appropriate indicators to measure this complexity? Could any composite index or a system of indices be developed that captures the multivariate interactive nature of the changing agriculture production needs dictated by an effective food systems transformation? Research to answer these questions is needed.

It will be important to maintain the momentum generated by the UNFSS and provide evidence-based approaches to successfully transform food systems.

## Funding

No funding was used for the development of the editorial.

## Conflict of interest

Neither author has any conflict of interest to declare.

## References

[1] HLPE, Nutrition and food systems (2017).

[2] GNR (2021).

[3] Micha R., Peñalvo J.L., Cudhea F., Imamura F., Rehm C.D., Mozaffarian D. (2017). Association between dietary factors and mortality from heart disease, stroke, and type 2 diabetes in the United States. JAMA.

[4] IHME, Global Burden of Disease 2021 (2024).

[5] UN (2024). United Nations: The 17 Goals.

[6] WHO, FIUW (2022).

[7] UN (2021). The Food Systems Summit [Internet].

[8] Masters WA, Finaret A.B. (2024). Food Economics: Agriculture, Nutrition and Health.

[9] Drewnowski A. (2010). The cost of US food as related to their nutritive value. Am. J. Clin. Nutr..

[10] Hendriks S., Groot Ruiz A.d., Acosta M.H., Baumers H., Galgani P., Mason-D'Croz D. (2021).

[11] The Rockefeller Foundation (July 2021).

[12] Tewodros T., Escobar C.X., Berra L.S., Girard A.W. (2024). Effectiveness of elements of social behavior change activities in nutrition-sensitive agriculture programs: a systematic review. Curr. Dev. Nutr..

[13] Webb P., Kennedy E. (2014). Impacts of agriculture and nutrition: nature of the evidence and research gaps. Food Nutr. Bull..

[14] Low J.W., Thiele G. (2020). Understanding innovation: the development and scaling of orange-fleshed sweetpotato in major African food systems. Agric. Syst..

[15] Lingala S., Freymond M., Tshering P.P., Kumari P., Kraemer K., Beesabathuni K. (2024). The Egg Hub Model: a sustainable and replicable approach to address food security and improve livelihoods. Curr. Dev. Nutr..

[16] Herforth A.W., Ballard T., Rzepa A. (2024). Development of the diet quality questionnaire for measurement of dietary diversity and other diet quality indicators. Curr. Dev. Nutr..

